# Spin hall effect of light ellipsometry for nanoscale areal surface measurement

**DOI:** 10.1038/s41598-025-95988-7

**Published:** 2025-04-15

**Authors:** Naila Zahra, Yasuhiro Mizutani, Tsutomu Uenohara, Yasuhiro Takaya

**Affiliations:** 1 Department of Mechanical Engineering, The University of Osaka, 2-1 Yamadaoka, Suita, Osaka 565-0871 Japan; 2 Instrumentation, Control, and Automation Research Group, Faculty of Industrial Technology, Institut Teknologi Bandung, Ganesa 10, Bandung, West Java 40132 Indonesia

**Keywords:** Engineering, Optics and photonics, Optical metrology

## Abstract

This paper presents a novel method for the measurement of nanometer-scale surfaces. The proposed technique takes advantage of the spin hall effect of light (SHEL), which occurs as a sub-wavelength beam shift due to the spin-orbit interaction of light when it interacts with non-homogeneous optical media. Governed by the conservation of total angular momentum, the SHEL offers a sensitive approach to detecting the variations of optical properties at an interface. “SHEL Ellipsometry” applies weak measurement principles to observe beam shifts, analogous to traditional ellipsometry, which analyzes the polarization states of incident and reflected light. In ellipsometry, a homogeneous sample with surface roughness less than a tenth of the wavelength can be modeled as a thin film characterized by an equivalent thickness and refractive index. By measuring the transverse shifts of the reflected beam and using raster scanning, SHEL Ellipsometry can map the two-dimensional surface roughness distribution, showing significant potential for nanometer-scale surface measurement.

## Introduction

The development of nanotechnology has revolutionized many fields, including the fabrication of nanoscale surfaces ranging from super-smooth surfaces to intricately structured surfaces. In high-precision optics, super-smooth surfaces are essential to reduce reflection and scattering losses^1^. Considering that, the development of the polishing technique has drastically improved up to the angstrom precision of roughness^2,3^. At the same time, nanostructured surfaces fabricated by various lithography techniques allow the development of a material with specific functionalities that cannot be achieved by existing natural materials^4,5^. In surface studies, at least two aspects become major concerns: the sufficient technology of fabrication or polishing and the corresponding measurement method^6^. Despite the significant progress in fabrication technologies, both in achieving angstrom-scale smoothness and complex nanostructures, the development of nanoscale surface instruments has lagged.

There are several instruments for nanoscale surface inspection, such as Atomic Force Microscope (AFM), Scanning Electron Microscope (SEM), white light interferometry (WLI), optical scatterometry, and ellipsometry. AFM is known to get a topographical map of a sample with sub-nanometer resolution by measuring the force between a cantilever tip and the surface under observation^7^. Obtaining information through AFM is rather time-consuming, especially considering the area under observation is limited to typically up to 100 x 100 micrometers. Besides that, the AFM probe tip is the major source of measurement uncertainty^8^. SEM is also known for surface roughness measurement^9,10^. However, the pre-coating for conductivity and the nature of the electron beam radiation leads to a limited observable sample and can be destructive to the sample. On the other hand, white light interferometry can obtain the whole area of the sample in a shorter time, which is interesting for an industrial inspection, such as for silicon wafer inspection^11^. However, WLI has limited lateral resolution, requires a reference surface, and the sample’s surface gradient introduces error in measurement^12^. AFM, SEM, and WLI are examples of direct measurement techniques to obtain the surface profile from physical interaction or direct imaging. In contrast, scatterometry and ellipsometry are indirect methods that extract surface information based on analysis of light-material interaction. Both are widely known instruments used for high-volume inspection of nanoscale surfaces^13^. In scatterometry, a structured surface is identified by calculating the diffraction efficiency derived from the measured diffraction intensity, which can be angle-dependent or wavelength-dependent^14^. Meanwhile, in ellipsometry, surface characterization is conducted by measurement of the polarization state of light as it interacts with the observed material^15^. Both techniques indirectly infer the surface and material properties of the illuminated area, making them beneficial for fast large-scale measurements of nanoscale surfaces, which are well-suited for industrial applications.

Priorly, ellipsometry is known as an instrument for film thickness and refractive index measurement with a high degree of precision and accuracy. Then, it is found to have a great sensitivity to surface roughness^16,17^. The surface roughness identification in ellipsometry serves two purposes, as correction to get the pure physical parameter without roughness influence and to differentiate different levels of surface roughness^18^. It has also been used complementary with other measurements such as AFM for surface characterization^13,19^. There are many types of ellipsometry based on its configuration and light source, and while it is a long-established measurement technique, it continues to evolve rapidly. The basis of ellipsometry is the measurement of the polarization state of light instead of intensity information that leads to its reliability and high accuracy^15,17^. Using ellipsometry, a tenth of wavelength roughness can be modeled as an ideal flat substrate with an equivalent thickness and optical property as a composite of the void and bulk material^17^. However, depending on its configuration of using various kinds of rotating modulators, unwanted systematic errors might likely arise, for example, due to unintended modulation and variation of temperature^15,20^.

In the last decade, there has been a growing interest in incorporating the Spin Hall Effect of Light (SHEL), which is one of subwavelength beam displacement due to the gradient of refraction index, for precision measurement. Previously, several applications of SHEL for precision metrology have been carried out to measure the film thickness, change of refractive index, optical constant, and even temperature^21–25^. In addition, SHEL observation is versatile, allowing it to study a wide range of materials, including transparent media, metals, and metasurfaces^26–29^. SHEL is transverse beam displacement that happens proportional to the working interface. Initially, it was a challenging task to observe the SHEL shift. However, since the demonstration of weak measurement to observe SHEL^26^, it has become easier to study the behavior and the application of this confined beam anomaly. SHEL observation with weak measurement is realized by defining the polarization state of the incident and reflected beam in a way similar to ellipsometry measurement. Also, similar to scatterometry and traditional ellipsometry, the optical properties of an observed surface can influence the measured transverse shift, providing insights into the surface characteristics. By incorporating SHEL with weak measurement and using the effective medium approximation (EMA) as a data analysis tool, SHEL ellipsometry has the potential as an imaging tool that can give nanoscale surface information.

This paper offers a novel nanoscale surface characterization using the SHEL ellipsometry. The measurement is done by measuring the SHEL shift of reflected light from the sample in an oblique incidence setup. The SHEL shift is amplified by the weak measurement and recorded using a CCD camera. A two-dimensional reconstruction of the surface was also obtained through raster scanning. In this proposed SHEL ellipsometry, no phase retarder is used, which completely avoids errors sourced from the retarder’s rotation or temperature dependence, further offering good stability for long-term measurement. For analysis, two different interface models are introduced: based on the Fresnel model that returned the parameter as pseudo refractive index and based on the effective medium approximation to obtain equivalent effective thickness. For accuracy assessment, a comparison of pseudo refractive index and known refractive indices of several optics is presented as a result of static single-point measurements. Furthermore, comparative scanning results are also presented to show the potential of SHEL ellipsometry as a novel nanoscale surface measurement.

## Principle of SHEL ellipsometry

In optics, the light interaction in the plane interface-whether reflection or refraction-is one of the fundamental processes generally governed by Snell’s law and the Fresnel equations. However, these conventional geometrical optics definitions of light evolution at the interface are insufficient for a confined beam with finite width, such as a Gaussian beam. Recent studies have revealed that the existence of wavelength scale shifts, known as the in-plane Goos-Hanchen shift^30^ and the out-of-plane Imbert-Fedorov shift^31,32^, add complexity to the light behavior at interfaces. These shifts can manifest as in-plane and out-of-plane spatial displacements relative to the incident plane and can also emerge as angular deflection. The out-of-plane transverse shift, known as the Imbert-Fedorov shift, was initially observed on the total internal reflection of circularly polarized light^31,32^. Then, it is known to also happen in partial reflection and has originated from spin-orbit interaction and is governed by the total angular momentum conservation known as the Spin Hall Effect of Light (SHEL)^33^. SHEL is closely associated with the polarization of light evolution and the properties of the interface^34^.

**Fig. 1:**
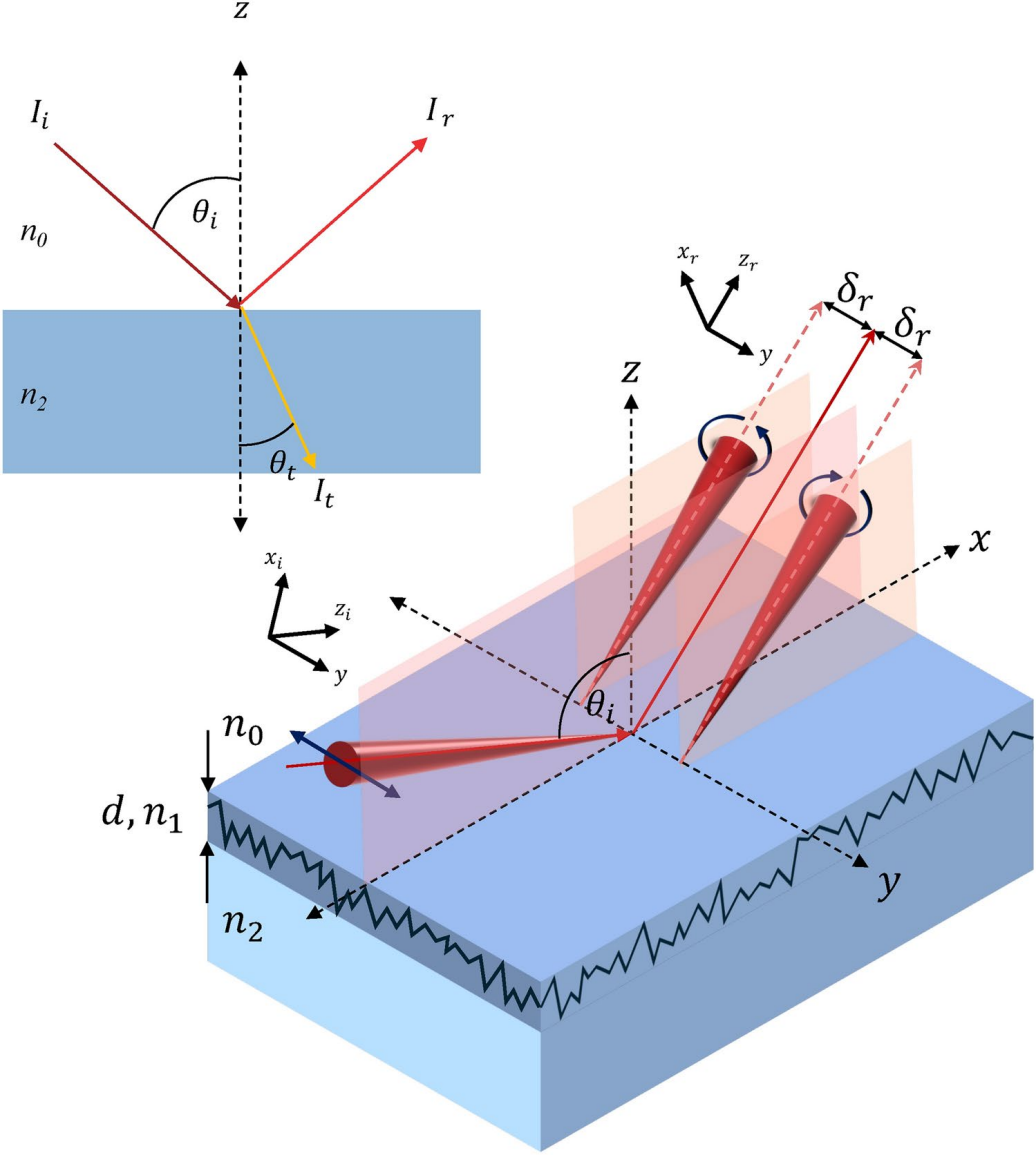
Principle of the spin hall effect of light at an interface, a linearly polarized incident beam will be reflected with different direction of circularly polarized light and transversely shifted at a distance of $\delta _r$. The 3D image was created using Microsoft PowerPoint version 16.93.2, Microsoft Corporation, (https://www.microsoft.com/powerpoint).

Light is known to have angular momentum which are the Spin Angular Momentum (SAM) related to the circular polarisation of light, the intrinsic orbital angular momentum (IOAM) related to the helical phase of beam, and the extrinsic orbital angular momentum (EOAM) characterizing beam trajectory^34^. Intriguing phenomena arise from the interaction of angular momentum; one of these is the spin-orbit interaction, which stimulates the Spin Hall Effect of Light, considering the interaction between the SAM and the EOAM^28^. The SAM is characterized by the degree of circular polarization $s=\pm 1$ for right-handed and left-handed polarisation^35^. The SHEL happens when the beam carrying spin angular momentum (SAM) incident at an angle $\theta _i$ on an interface with a gradient of index refraction leads to the transformation of SAM to external orbital angular momentum, which manifests in terms of beam deflection from its supposed trajectories. This polarization state evolution and beam propagation in inhomogenous media is also related to the geometric phase.^33^ The incident beam having linear polarization will be reflected as a split beam with circular polarization with different handedness^36^. As shown in Fig. 1, when a linearly polarized beam is incident at $\theta _i$, the two circularly polarized beams are separated at an equal distance of $\delta _r$ from the incidence plane. The balance of total angular momentum governs this SHEL phenomenon, and the shift corresponds to the polarization state of the incident and reflected beam^33^.

1 $$\begin{aligned} \delta _r = \frac{\langle \Psi _r | {\hat{\sigma }}_z | \Psi _r \rangle - \langle \Psi _i | {\hat{\sigma }}_z | \Psi _i \rangle }{k_i \tan {\theta _i}} \end{aligned}$$

Based on Eq. (1), measuring the SHEL shift $\delta _r$ can be done when the state of polarization of incident $\Psi _i$ and reflected beam $\Psi _r$ as well as the incident angle $\theta _i$ are known, with ${\hat{\sigma }}_z$ is the Pauli spin matrix and $k_i$ is the wave number. It opens the possibility of a new kind of ellipsometry-like measurement. Furthermore, for incident light with horizontal and vertical polarization, SHEL shift reflection can be quantified based on the interaction with the medium as Eqs. (2) and (3), respectively^37^.

2 $$\begin{aligned} \delta _{H|{\pm }\rangle } = {\mp } \frac{1 + \frac{r_s}{r_p}}{k_i} \tan {\theta _i} \end{aligned}$$

3 $$\begin{aligned} \delta _{V|{\pm }\rangle } = {\mp } \frac{1 + \frac{r_p}{r_s}}{k_i}\tan {\theta _i} \end{aligned}$$

With $r_p=|r_p|e^{i\phi _p}$ and $r_s=|r_s|e^{i\phi _s}$ being the complex Fresnel reflection coefficients of the interface for the $p$ and $s$ polarization, respectively. Both Eqs. (2) and (3) contain the Fresnel reflection coefficient ratio, which inherently carries information about the interface. Therefore, any change in the physical properties at the interface will be reflected as a change in the Fresnel reflection coefficients.

It is widely known that the Spin Hall Effect of Light (SHEL) shift amount is on the sub-wavelength order $\delta _r \approx \lambda / 2\pi$^34^, which makes observation challenging. The most widely used method for observing sub-wavelength beam shifts is weak measurement, which have been employed to observe not only the SHEL shift but also the Goos-Hänchen shift and the Imbert-Fedorov shift^26,38,39^. Through weak measurement, a complex weak value amplifies the weak interaction through the predetermined initial and final states. In the case of SHEL shift measurement, the initial and final states correspond to the selection of the light polarization state upon incidence and reflection.

**Fig. 2:**
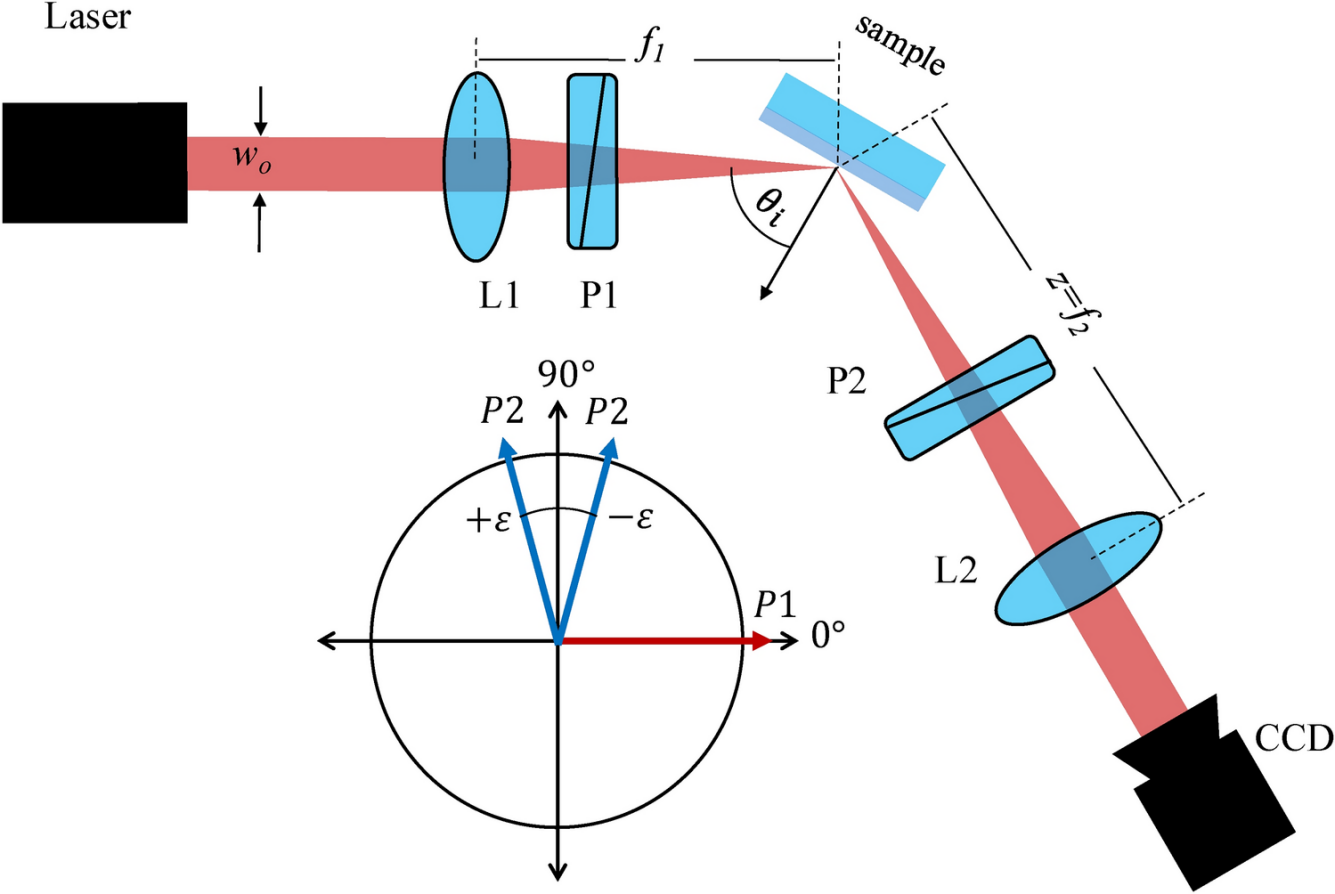
Schematic of SHEL ellipsometry, (inset) polarizer state corresponding to the weak measurement’s requirement.

Figure 2 shows the schematic setup for SHEL ellipsometry based on SHEL observation using weak measurement. Polarizer pairs realize the weak measurement through the definition of light polarization as the initial $|i\rangle$ and final state $|f\rangle.$

4 $$\begin{aligned} A_w^n= \frac{\langle f | {\hat{A}}^n | i \rangle }{\langle f | i\rangle } \end{aligned}$$

$A_w^n$ is the $n$th order weak value, ${\hat{A}}^n$ is the weak coupling representing the weak interaction^40^. In the case of SHEL measurement, it corresponds to the Pauli spin matrix in the $z$-direction^41^. Based on Eq. (4), the weak value is largest when the final and initial states are almost orthogonal. For weak measurement setups, a small azimuth angle denoted by $\varepsilon$ is commonly introduced in the final state. For example, if the incident beam is set to have horizontal polarization $|H\rangle$, then the final state is $|V + \varepsilon \rangle.$

The amplified SHEL shift observed is defined as $\delta _A = F |A_w| \delta _r$, which shows that the observed amplified SHEL is the amplification of the original SHEL shift by the weak value $A_w$ and the free propagation factor $F$^26,42^. $F$ is related to $z$ the effective propagation distance and the Rayleigh length $z_R$^43^. In this paper, this model is called the first-order model due to the first-order Taylor series expansion of the unitary operator used. Replacing $\delta _r$ with Eq. (2) and representing the Fresnel ratio $r_p/r_s$ as variable $\rho$, the first-order amplified SHEL shift for a horizontally polarized incident is denoted by:

5 $$\begin{aligned} \delta _{A1}^{H|{\pm }\rangle } = {\mp }\frac{f_2 w_{o}^2 (1 + \rho ^{-1})}{8 f_1^2 \tan \varepsilon \tan \theta _i} \end{aligned}$$

where $f_1$ and $f_2$ correspond to the pair of lenses in Fig. 2 and $w_{o}$ is the beam diameter at the laser outlet that relates to the propagation factor of the SHEL shift.

Equation (5) works well for a horizontally polarized incident beam only when the incident angle is far from the sample’s Brewster’s angle. Near Brewster’s angle, an enormous shift should occur based on Eq. (5), but it disagrees with the actual conditions, leading to numerous modifications of the model^42,44,45^. There are several approaches to improve the model inaccuracy in the vicinity of Brewster’s angle for the $|H\rangle$ incident, for example, by performing Taylor expansion of the reflection coefficient or by improving the weak measurement model. This time, the modified weak measurement is applied, and the amplified SHEL shift for $|H\rangle$ incident is denoted by:

6 $$\begin{aligned} \delta _{A2}^{H|{\pm }\rangle } = {\mp }\frac{2f_2(1 + \rho ^{-1})\tan \varepsilon \tan \theta _i w_{o}^2}{(1 + \rho ^{-1})w_{o}^2 + 16f_1^2 \tan ^2 \varepsilon \tan ^2 \theta _i} \end{aligned}$$

Similar to other forms of ellipsometry, SHEL ellipsometry relies on comprehensive modeling of the system under observation for data interpretation. The variable $\rho$, is crucial for retrieving the physical parameters of the object. Conventionally, $\rho =tan\psi e^{i\Delta }$ are retrieved from ellipsometry measurement with $\psi$ and $\Delta$ known as ellipsometry angles, which are then calculated into physical parameters based on the assumed model in the measurement^46,47^. In SHEL ellipsometry, once the amplified SHEL shift is observed and the experimental parameters are known, $\rho$ can be obtained. Then, based on the assumed interface model, $\rho$ is used to calculate the physical parameters. The simplest model of an interface in optics is the air-glass interface, where light propagates from air to a more condensed medium, resulting in a change of refractive index along the propagation trajectory. In this work, the objects are non-absorbing optics, and $\rho$ is defined using Snell’s Law and the Fresnel formulas as:

7 $$\begin{aligned} \rho = \frac{r_p}{r_s} \end{aligned}$$

The air-glass interface is assumed to be ideal, without any roughness, and $\rho$ is simply defined as the ratio of Fresnel reflection coefficients *p* over *s*, referred here as the Fresnel model. Referring to the sample in Fig. 1, the refractive index of the air is $n_0$ while the ideally smooth sample’s refractive index is $n_2$, which is retrieved from SHEL measurements and is also addressed as the “pseudo” refractive index.

As commonly used in ellipsometry, roughness less than a tenth of a wavelength can be modeled using the effective medium approximation (EMA), where the roughness layer is assumed as an equivalent thin film with its optical properties and effective thickness that represents the rms roughness^48,49^. In this research, we use the Bruggeman effective medium approximation to estimate the refractive index of the roughness layer. Using the Bruggeman EMA, given the refractive indices of air and sample then the refractive index of the roughness layer can be calculated as the composite of air and the bulk sample layer with a volume fraction defining the composition ratio^18,50^. The volume fraction is commonly set as 0.5 to define the roughness layer’s refractive index, then the roughness equivalent thickness can be assumed to represent the real surface^49^. By using this approach, the interface becomes an air-roughness layer-sample, and $\rho$ can be defined through multi-layer reflection in Eq. (8).

8 $$\begin{aligned} \rho = \frac{(r_{1,p} + r_{2,p} \chi )(1 + r_{1,s} r_{2,s} \chi )}{(r_{1,s} + r_{2,s} \chi )(1 + r_{1,p} r_{2,p} \chi )} \end{aligned}$$

where $\chi = \exp \left( -\frac{4\pi d_{EMA} n_1 \cos \theta _i}{\lambda }\right)$, $d_{EMA}$ being the roughness equivalent thickness, and $n_1$ the refractive index of the roughness layer from Bruggeman EMA as illustrated in Fig. 1. The number subscript in Eq. (8) defines the air-thin layer and thin layer-bulk interfaces, respectively. Therefore, from the observation of the SHEL shift, the surface information can be retrieved by solving Eq. (6) based on the appropriate model of ellipsometry parameters, either as the pseudo refractive index using Eq. (7) or as the roughness equivalent thickness based on Eq. (8).

## Numerical calculation of SHEL ellipsometry

**Fig. 3:**
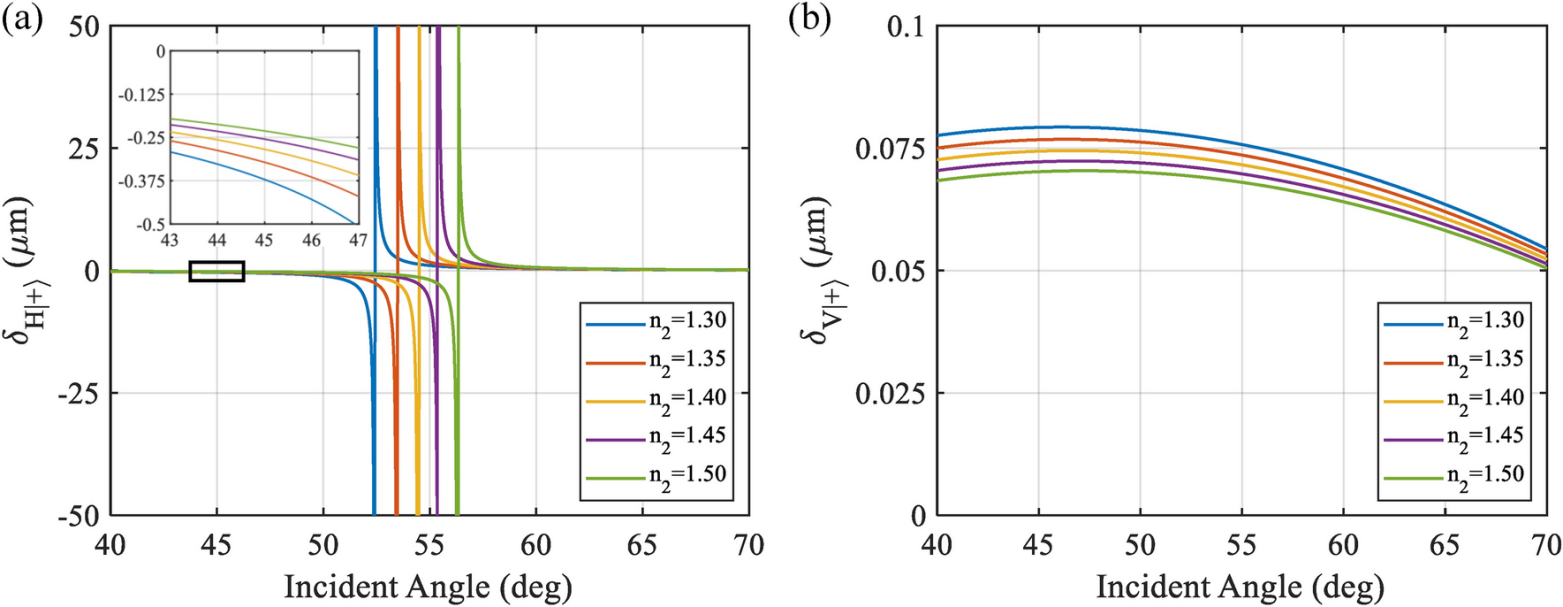
Numerical calculation of SHEL shift to incident angle on different refractive index (**a**) horizontally polarized incident beam and (**b**) vertically polarized incident beam.

A numerical simulation was conducted to confirm the theoretical model of SHEL ellipsometry. Figure 3 shows the original SHEL shift reflected from the air-glass interface with various refractive indices varying from 1.30 to 1.50 for horizontally polarized incident beam and vertically polarized incident beam based on Eqs. (2) and (3). From Fig. 3a, it can be seen that the amount of shift for the horizontally polarized incident beam is significantly larger than the vertically polarized incident beam shown in Fig. 3b. This shows how the horizontal incident can be more beneficial for measurement compared to the vertically polarized incident beam. Furthermore, for a horizontally polarized incident beam, the shift is symmetrical and changes sign at a special incident angle where it coincides with the sample’s Brewster s angle, $\theta _i=\theta _B$, where $\theta _B=tan^{-1}\frac{n_2}{n_0}$. In the vicinity of $\theta _B$, the shift grows larger, opening the possibility to be taken advantage of. However, measurement at $\theta _i=\theta _B$ is impossible to conduct as the $r_p\rightarrow 0$ which makes Eq. (2) undefined. From a weak coupling point of view, the condition near the $\theta _B$ is a strong coupling. The nature of horizontally polarized incident with the $\theta _B$ also shows how it is sensitive to the refractive index at the interface. In contrast, from Fig. 3b for the vertically polarized incident beam, all incident angles can be used without any anomaly since there is no special angle $\theta_B$. The interaction of vertically polarized incident beam is a weak coupling for all incident angles^42^. However, the shift range is limited, which may not benefit the measurement process. Moreover, even after amplification, the tiny shift in the vertically polarized incident beam might require a higher resolution CCD to get a precise SHEL shift. Otherwise, it would be more prone to error.

**Fig. 4:**
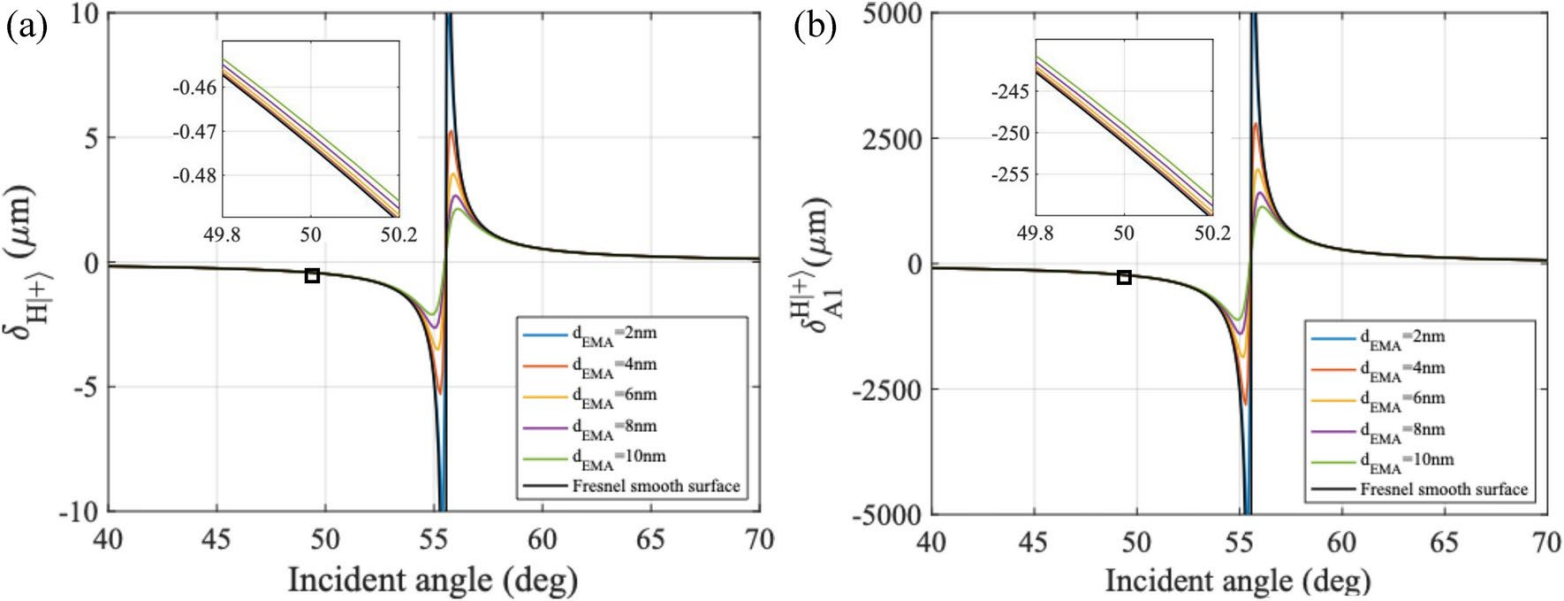
Numerical calculation for the horizontally polarized incident on SiO$_2$ flat windows with various levels of roughness d$_{EMA}$ (colored lines) compared to Fresnel smooth surface (black line) (**a**) original SHEL shift and (**b**) amplified SHEL shift due to weak measurement based on Eq. 5.

Figure 4 shows the SHEL shift and amplified SHEL shift subject to the two interfaces due to the medium approximation of the roughness layer. Varying roughness equivalent thickness was calculated from 2 nm to 10 nm and compared to the Fresnel model of air-glass interface that shows 0 nm roughness. Here, only the horizontally polarized incident beam is observed. Figure 4a shows how different effective thicknesses affect the SHEL shift value. This indicates that the SHEL shift information would change when the medium interface varies. In this case, the surface roughness contributes to the change in the refractive index of the sample. Figure 4b shows a calculation of the amplified shift to incident angle due to the weak measurement with the post-selected angle assigned for this calculation is $\varepsilon =1.5^\circ$ and the focal length of 100 mm and 175 mm for L1 and L2, respectively. Comparing the two plots shows the significance of the amplification for the measurement. These calculations show the potential of using the information of SHEL shift to observe nanoscale surface roughness.

## Result and discussion

### Experiment condition

**Fig. 5:**
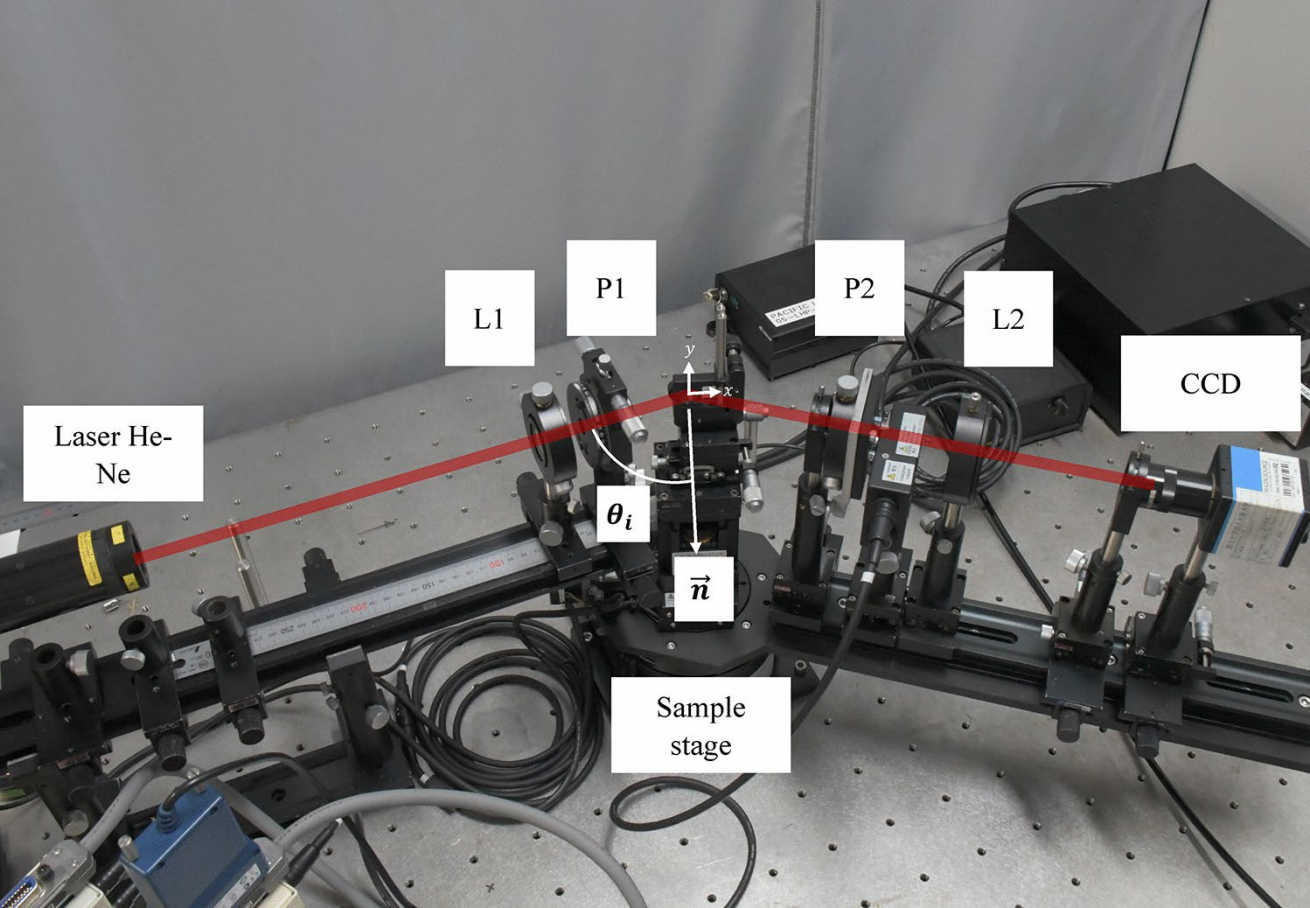
Optical setup of SHEL ellipsometry.

Figure 5 shows the experiment setup of SHEL ellipsometry. Three parts can be seen which are the incident arm, the sample stage, and the detection arm. Similar to the schematic in Fig. 2, the illumination source is a He-Ne laser $\lambda$ =632.8 nm, producing a linearly polarized Gaussian beam. As mentioned, the polarizer pair is used to realize the weak measurement setup. A polarizer in the incident arm decides the initial state P1 of the incident light. A lens with a focal length of 100 mm focused the beam to the sample. In the detection arm, a polarizer defined the final state P2 set $90 \pm \varepsilon$ from the initial state P1. Then, the second lens (L2), with a focal length of 175 mm, collimates the reflected beam to the CCD camera that records the beam shift. The CCD parameters, such as gain and exposure time, remain unchanged for all measurements to ensure experiment consistency. The sample stage is mounted on a double rotation mounting that rotates not only the sample stage but also the detection arm to set the incident angle while the incident arm is static all the time. Besides that, the sample stage is also mounted on top of the $xy$ translation stage, and the sample is moved in the scanning mode. In other research works, the setup for SHEL observation using weak measurement commonly includes a half-wave plate between the laser and the first lens to help with the intensity modulation by rotating the polarization direction^23,26^. In this work, no phase retarder is included in the setup to completely avoid the source of error from the retarder. Alignment of the direction of laser polarization and the P1 axis is ensured instead of adding any retarder.

The experiment procedure is started by setting the sample stage and detection arm to form the desired incident angle. After that, the pair polarizer is set exactly perpendicular to each other or in a cross polarizer state, without the small $\varepsilon$ angle in P2. This state is essential to observe the splitting beam condition where two symmetrical intensities of SHEL beams appear. The dark fringe between the two intensities is defined as the datum for measuring the SHEL shift. After that, by using the motorized rotation mount, the $\varepsilon$ is added to the P2 to start the weak measurement. Depending on the desired information, both static measurement and scanning measurement can be performed. The scanning measurement is performed by raster scanning through moving the $xy$ translation stage.

### Single-spot static measurement

**Fig. 6:**
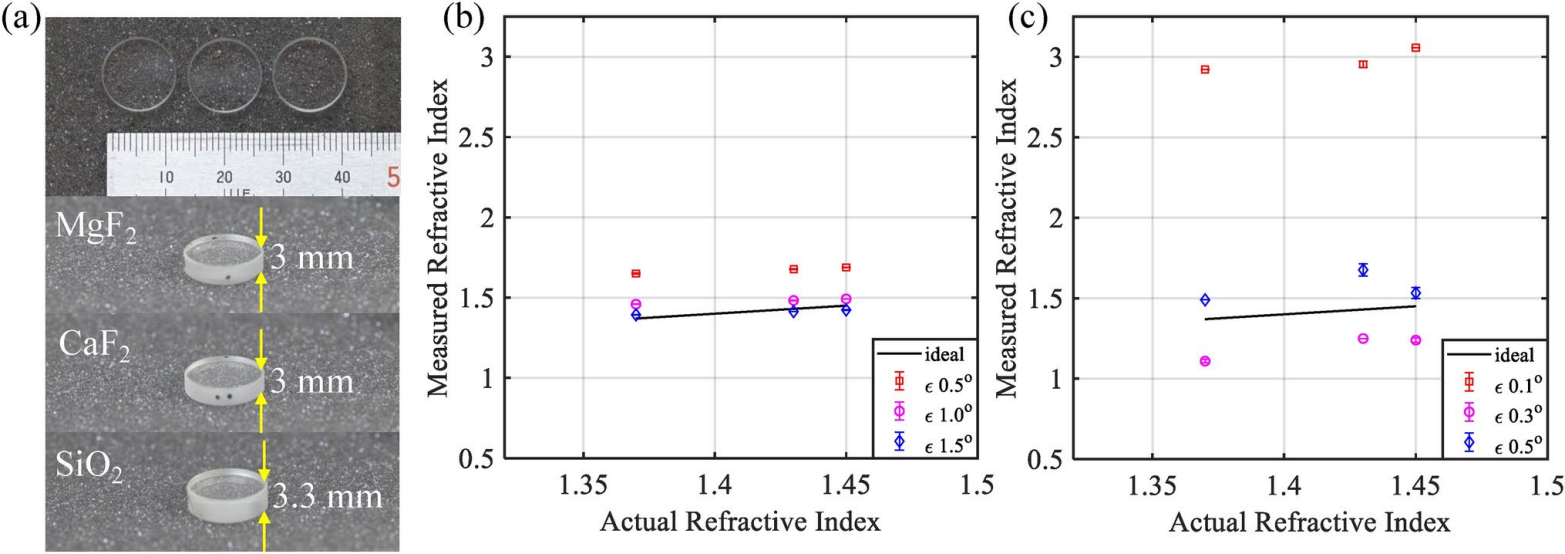
Accuracy check by single point pseudo refractive index measurement of (**a**) samples consisting of flat windows of different material MgF$_2$ ($n = 1.37$), CaF$_2$ ($n = 1.43$), SiO$_2$ ($n = 1.45$), (**b**) measurement result of horizontally polarized incident beam, (**c**) measurement result of vertically polarized incident beam.

The following experiment is a SHEL ellipsometry measurement to retrieve the refractive index of glass or the pseudo-refractive index. Three windows made from different materials with known refractive index from their specifications were observed: MgF$_2$ ($n = 1.37$), CaF$_2$ ($n = 1.43$), and SiO$_2$ ($n = 1.45$). The windows’ diameters are 1 inch, with thicknesses of 3 mm and 3.3 mm, as shown in Fig. 6a. Static measurements were carried out with 30 data points for each parameter. The incident angle for all the measurements was kept at $\theta _i = 50^\circ$. For the $|H\rangle$ incident, the post-selected azimuth angles were $0.5^\circ$, $1.0^\circ$, and $1.5^\circ$, while for the $|V\rangle$ incident, the post-selected azimuth $\varepsilon$ was $0.1^\circ$, $0.3^\circ$, and $0.5^\circ$. The pseudo-refractive index was retrieved based on the air-glass model on Eq. (7). The measurement results for the $|H\rangle$ incident and $|V\rangle$ incident are shown in Fig. 6b and c, respectively.

As can be seen, the results from the $|H\rangle$ incident measurements are more accurate compared to the known values than those from the $|V\rangle$ incident measurements. The trend of the measurement results using $|H\rangle$ also shows agreement with the actual value of the refractive index. On the other hand, the $|V\rangle$ incident with $\varepsilon = 0.3^\circ$ and $0.5^\circ$ shows a false trend and fails to retrieve the correct value of the refractive index. The $|V\rangle$ incident is more susceptible to error due to the small value of shift. Therefore, the shift difference due to varying medium properties will be even smaller, leading to more difficulty in observation. Although the $|V\rangle$ incident is inherently not limited by Brewster’s angle of the sample, the tiny range of shift value in this proposed setup makes it a less favorable option. This experiment demonstrates the potential of SHEL ellipsometry in retrieving the physical properties of samples, as confirmed by comparing the pseudo-refractive index to the known refractive index of the samples.

For the $|H\rangle$ incident beam, it can be seen that as the post-selected angle increases, the accuracy also improves and becomes closer to the ideal trend line. A static measurement was carried out for varying incident angles to further understand the SHEL observation using weak measurement, both from the perspective of the weak measurement model and from the measurement parameter. The incident angle for the observation was varied by $1^\circ$ increments from $40^\circ$ to $70^\circ$. Besides the space limitation due to the component size, which prevents smaller incident angles, the focus of the measurement was on incident angles closer to Brewster’s angle. In this experiment, a SiO$_2$ optical flat with a refractive index of 1.45 ($\theta _B= 55.5^\circ$) was used. The post-selected azimuth angles $\varepsilon$ were $0.5^\circ$, $1.0^\circ$, and $1.5^\circ$.

**Fig. 7:**
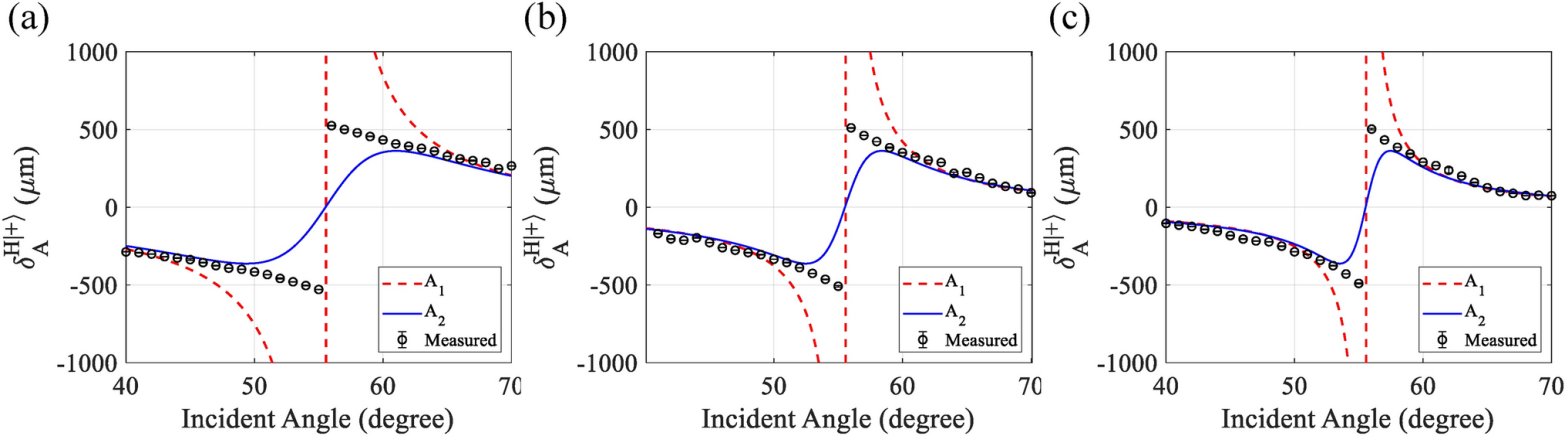
Measurement of amplified SHEL shift reflected from SiO$_2$ ($n = 1.45$) compared to different model of weak measurement model, red dash line represents first order weak measurement model, solid blue line represents the second order weak measurement, and black circle represents measured shift at each incident angle from 40$^\circ$ to 70$^\circ$ (**a**) azimuth angle 0.5$^\circ$, (**b**) azimuth angle 1.0$^\circ$, (**c**) azimuth angle 1.5$^\circ$.

Figure 7a–c shows the measurement results compared to the theoretical values for azimuth angle 0.5$^\circ$,1.0$^\circ$,1.5$^\circ$, respectively. The red dashed line represents the 1st-order weak measurement model, the blue solid line represents the 2nd-order weak measurement model, and the black circle is the measured shift value. The cross-section between the amplified shift with the $x=0$ indicates the location where the incident angle coincides with Brewster’s angle. It can be seen that $\delta _{A1}^{H|+\rangle }$ grows very large in the vicinity of Brewster’s angle and that the measured amplified shift does not agree with this model. On the contrary, the measured amplified shift agrees better with the calculated $\delta _{A2}^{H|+\rangle }$ until the very narrow region before and after $\theta _B$.

The second point to note from this experiment is the influence of the post-selected azimuth angle ($\varepsilon$). For $\delta _{A1}^{H|+\rangle }$, with larger $\varepsilon$, the incident angle range in which the amplified shift grows extremely large becomes narrower, or closer to $\theta _B$. For $\delta _{A2}^{H|+\rangle }$, the inclining turnaround line, where the shift changes from negative to positive, becomes sharper with the selection of larger $\varepsilon$. With the steeper trend of $\delta _{A2}^{H|+\rangle }$, the point of agreement between the measured amplified shift and the calculated $\delta _{A2}^{H|+\rangle }$ shows a wider range of incident angles that can be chosen for measurement. Additionally, given that the SHEL sensitivity is higher in the vicinity of Brewster’s angle, a higher azimuth angle is preferable to achieve a good measurement when selecting an incident angle closer to $\theta _B$. This experiment explains the affecting parameter of the measurement result in Fig. 6 and guides the setup parameter decisions for the measurement, the incident angle ($\theta _i$) and the post-selected azimuth angle ($\varepsilon$).

### Scanning measurement

One of the main proposals of this work is the reconstruction of the two-dimensional surface profile of the object under observation through raster scanning. As shown in Fig. 5, the sample stage is mounted on a motorized $xy$stage, and data are acquired by recording the beam shift at every spot in the X and Y directions by moving the stage. To obtain good resolution, the scanning is conducted with overlapping steps smaller than the focused beam diameter. The focused beam waist in this setup is 101 $\upmu$m, and the scanning step for all measurements is 50 $\upmu$m in both the $x$ and $y$ directions. After obtaining the data from all the spots along the raster scanning trajectories, each data point is processed based on the SHEL ellipsometry model to retrieve the physical parameters, followed by the reconstruction of the 2D distribution.

**Fig. 8:**
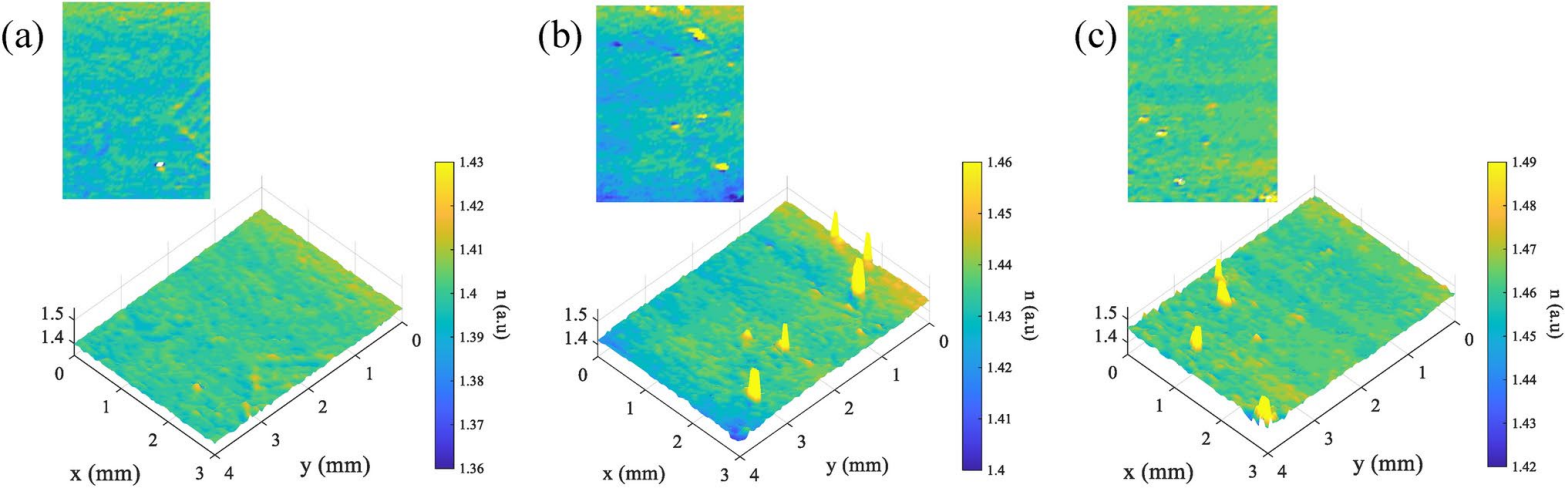
Scanning measurement of optics samples (**a**) MgF$_2$ ($n = 1.37$), (**b**) CaF$_2$ ($n = 1.43$), (**c**) SiO$_2$ ($n = 1.45$).

Figure 8a–c shows the scanning results of the same samples from Fig. 7, which are MgF$_2$, CaF$_2$, and SiO$_2$. The scanning was conducted with $\theta _i = 50^\circ$ and $\varepsilon = 1.5^\circ$. It is important to note that both $\theta _i$ and $\varepsilon$ are unaltered during scanning process. The scanning area for each sample is 3 mm $\times$ 4 mm. The reconstruction shows the retrieved pseudo-refractive index of the samples, as indicated by the color bar. The distribution of the retrieved pseudo-refractive index from the surface indicates the presence of a physical quantity causing its value to vary. From the plot in Fig. 8, the measurement shows interesting scratch-like features on the object that potentially originate from the actual condition of the sample. It is important to note that a feature larger than the nanoscale, whether it originates from the sample or debris on the sample, could create a high scattering that cannot be detected by SHEL ellipsometry. It usually appears as an outlier value, for example, it is shown as a yellow saturated value in Fig. 8. Besides examining the two-dimensional distribution, the average pseudo-refractive index consistently shows accuracy to the actual refractive index values: MgF$_2$ ($n = 1.37$), CaF$_2$ ($n = 1.43$), and SiO$_2$ ($n = 1.45$). SHEL ellipsometry measurement proposes a novel surface roughness measurement in terms of the pseudo-refractive index. Since the mean pseudo-refractive index can imply the general refractive index value, the variance of the pseudo-refractive index can represent the degree of roughness. Therefore, it can be beneficial for comparative roughness measurement of samples made from the same material with varying roughness information.

**Fig. 9:**
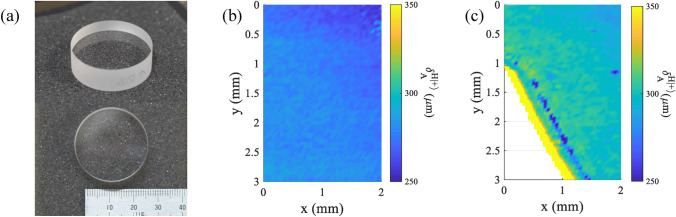
Scanning of different parts of optical flat (**a**) sample, (**b**) reconstruction of the central area, (**c**) reconstruction of edge area.

**Fig. 10:**
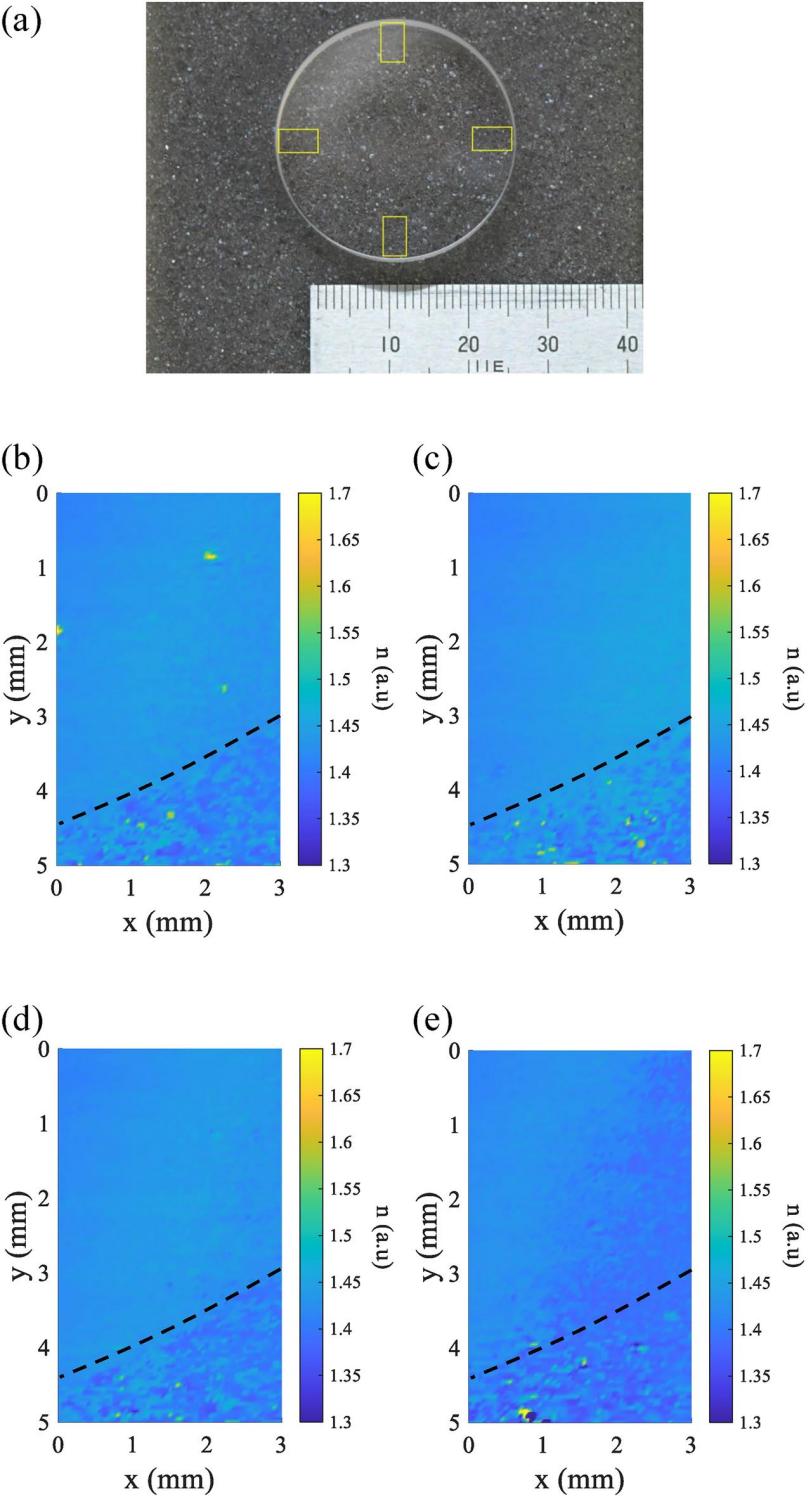
Scanning of the optical flat four quadrant’s edge shows different levels of roughness on the outer edge circle detected by SHEL ellipsometry: (**a**) Result on 1$^{st}$ area, (**b**) 2$^{nd}$ area, (**c**) 3$^{rd}$ area, and (**d**) 4$^{th}$ area.

The second scanning experiment was conducted on a different sample: a high-precision polished fused silica optical flat ($n = 1.45$) with a diameter of 30 mm and a thickness of 10 mm (Sigmakoki HMPQP-30C10-20), as shown in Fig. 9a. According to the specifications, it has a 95% clear aperture, meaning the remaining 5% may have imperfections or not meet the surface quality intended for application. Different degrees of roughness can be expected on the outer part; furthermore, as is common in optical elements, there is an edge indentation in the outermost part. A comparison between the center and the outer area was performed with the same experimental parameters as before, but the scanning area was 2 mm$\times$ 3 mm. Figure 9b and c show the measurement results of the center and edge areas, respectively. As can be seen, the center part shows a uniform tendency of shift value that implies a uniform roughness degree. On the contrary, for the edge area, higher varying shifts are acquired: the area in the top right corner is closer to the center part of the sample, then as it gets further to the edge, the amplified shift significantly varies, and the yellow strip on the bottom left corner of the reconstruction shows an extreme value that represents the rollover indentation part. The indentation part is very rough compared to the whole surface of the optical flats, leading to higher scattering. Following that, the scanning was conducted in the four quadrants of the optical flat with an area of 3 mm $\times$ 5 mm, as shown by yellow squares in Fig. 10a. Figure 10b–d shows the reconstruction of the four quadrant areas. The reconstructions show similar general features where there is different surface roughness between the center having relatively smooth features compared to the rough area in the bottom part with apparent transition marked by the black dash line. On the rough area, it can be observed how the measured refractive index shows more variability relative to the actual refractive index value of the samples. Not only that, lower values of the refractive index are also observed in the rough area, which could originate from the slight slope profile of the outer ring part compared to most area that leads to the incident angle error. From the experiments shown in Figs. 8, 9, 10, it can be inferred that SHEL ellipsometry scanning can retrieve the actual condition of the sample surface.

**Fig. 11:**
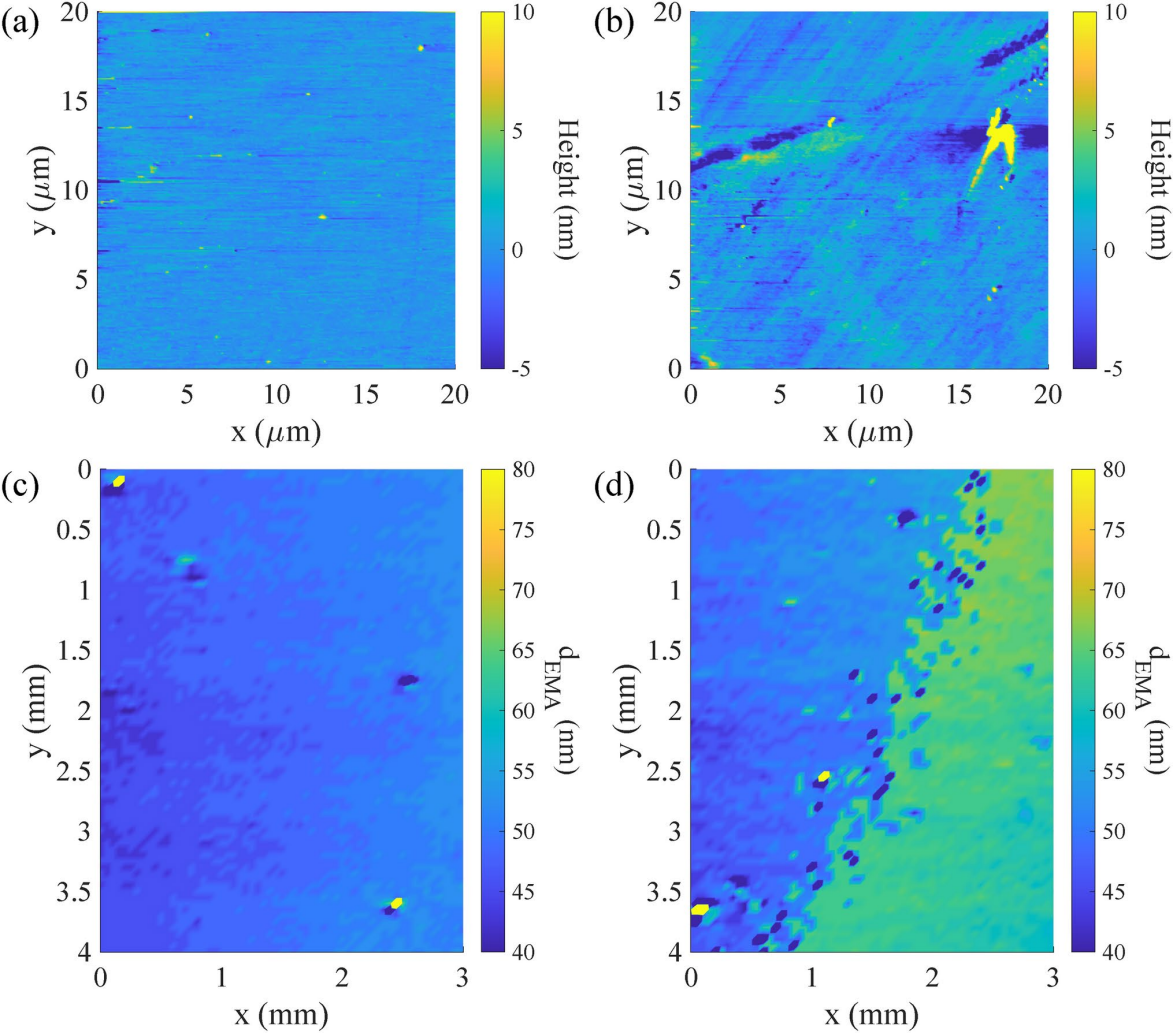
Comparison of polished and unpolished surface from AFM and SHEL ellipsometry (**a**) AFM of polished surface, (**b**) AFM of unpolished surface, (**c**) SHEL scanning of polished surface, (**d**) SHEL scanning of unpolished surface.

The previous measurement is relatively qualitative since there is no exact information about the actual value of the different levels of surface roughness. In this last experiment, we conducted a comparative scanning of two different surface roughness levels of the same material. One of the samples in Fig. 3a is a single-surface optical flat made of SiO$_2$ ($n = 1.45$, Edmund #43-400-000), meaning that the front and rear surfaces of the optical flat are different, as the front part is precisely polished while the rear part is not. To confirm this information, a measurement using an Atomic Force Microscope (AFM) was performed over a scanning area of 20 μm $\times$ 20 $\upmu$m with scan lines of 256. As shown in Fig. 11a and b, the polished surface and unpolished surface exhibit different roughness levels. Quantitatively, the polished part has a Sa of 0.605 nm, while the unpolished part has a higher Sa of 1.25 nm. The AFM measurement confirmed the different roughness levels of the front and rear parts.

Scanning SHEL ellipsometry was conducted on both sides of the optical flat over a 3 mm $\times$ 3 mm area. In the study of ellipsometry, $d_{EMA}$ is known to be proportional to the RMS roughness of the same area measured by a profilometer^49,51^. The SHEL ellipsometry reconstructions are shown in Fig. 11c and d for the polished and unpolished surfaces, respectively. The calculated areal surface roughness in terms of $d_{EMA}$ for the polished surface is 48.65 ± 6.73 nm, while the unpolished surface measures 55.89 ± 11.76 nm. The measurement result shows how the different polishing conditions of the sample’s surface inferred as the thickness equal roughness. The polished area has a lower $d_{EMA}$ compared to the unpolished one, which demonstrates the potential of scanning SHEL ellipsometry for surface roughness measurement. This technique, utilizing the EMA model, is particularly well-suited for samples with a known refractive index ($n_2$) for accurate analysis.

The measurement using SHEL ellipsometry can be considered an area-integrating method as the surface information retrieved from the measurement at every spot carries the integral information of a specific area under the beam exposure^52^. The comparison between AFM and the SHEL ellipsometry results indicates that SHEL ellipsometry can effectively discriminate polished and unpolished surfaces. However, an exact numerical comparison cannot be done because of the different principles of surface extraction involved^53,54^. It is important to note that a direct comparison between the AFM and SHEL ellipsometry should not be made due to the differing parameters and scanning areas involved where the SHEL ellipsometry scan covers a significantly larger area than the AFM scan^55^.

Accuracy, precision, and repeatability are essential in measurement. Experiments suggest proposed SHEL ellipsometry has good precision and repeatability through validation of refractive index measurement and consistency of roughness measurement with AFM. However, the lack of calibration standards for nanoscale roughness^56^ limits the direct assessment of the accuracy. Additionally, certain angle parameters in SHEL ellipsometry play crucial roles that impact amplification and measurement results, yet compensating for angle error is also challenging, contributing to the uncertainty. Future studies should be made to assess the accuracy, such as correlation analysis between AFM and SHEL ellipsometry across varying known surface roughness levels.

Our study indicates that SHEL observation using weak measurement is analogous to ellipsometry in that the polarization states of incident and reflected light are crucial. Since the polarizer pair in SHEL ellipsometry is not dynamically rotating and remains static during measurement, systematic error from the rotating retarder/analyzer will not be produced. Furthermore, with the absence of a phase retarder, SHEL ellipsometry completely avoids the temperature dependence of a phase retarder, making it stable for long measurements, as demonstrated by the scanning measurement. The flexibility of SHEL ellipsometry enables it to gather surface property information based on the medium’s model definition. For smooth surfaces assumption, these properties can be represented as a distribution of pseudo-refractive indices, while for rough surfaces assumption, the roughness can be expressed as effective thickness using the EMA. Additionally, the raster scanning capability of this proposed method allows for the reconstruction of surface conditions across the sample without limitations on the applicable area size. This study focused on samples without absorption. Nevertheless, its applicability could be improved to accommodate materials with absorption by applying the expanded model available from referenced literature^57,58^, as well as utilizing an arbitrary incidence polarization angle for greater flexibility.

The demand for subnanometer areal surface inspection is rapidly increasing with the advancement of materials, metasurfaces, and high-precision optical systems. This proposed instrument offers a simple hardware setup without sample size constraints while enabling nanoscale surface characterization. Although it does not provide direct measurements, its efficiency competes with established existing methods such as AFM and SEM. Moreover, the inferred surface information can be flexibly modeled to suit different sample types, allowing SHEL ellipsometry to analyze a broad range of materials. This adaptability makes it valuable for diverse applications, from precision glass polishing to metamaterial development.

## Conclusions

This paper demonstrates the application of the SHEL ellipsometry for areal surface measurement. Firstly, the confirmation of the model with the experiment shows a good agreement. Moreover, the influence of the post-selected azimuth angle on the measurement result is evident from the retrieval of the pseudo-refractive index. Secondly, the scanning SHEL ellipsometry shows the reconstruction of the nanometer feature of observed samples. Comparative measurement of known polished and unpolished surfaces validate SHEL ellipsometry as a novel roughness measurement technique, with roughness equivalent thickness $d_{EMA}$ representing different levels of roughness. Additionally, the roughness parameter can be expressed as either a pseudo refractive index or an equivalent roughness thickness, depending on the sample’s known properties. SHEL’s sensitivity to changes in the medium’s refractive index offers a significant advantage for nanoscale surface inspection. Moreover, with unlimited sample size and the ability to indirectly infer the properties of the illuminated area, SHEL ellipsometry enables rapid assessment over large area measurements. Validating the absolute measured value of surface roughness from SHEL ellipsometry presents a challenge, much like other nanometer-scale instruments. Future assessment methods could include correlation analysis or the development of calibration procedures and techniques.

## Data Availability

The data sets generated during the current study are available from the corresponding authors on reasonable request.
